# TOPSS: TOlerability of transcranial direct current stimulation in Pediatric Stroke Survivors

**DOI:** 10.3389/fnhum.2025.1629499

**Published:** 2025-08-05

**Authors:** Stuart Fraser, Anna Clearman, Melika Abrahams, Bernadette Gillick, Tia Lal, Sean Savitz, Nuray Yozbatiran

**Affiliations:** 1 Division of Child and Adolescent Neurology, Department of Pediatrics, McGovern Medical School, University of Texas, Houston, TX, United States; 2 Institute for Stroke and Cerebrovascular Disease, University of Texas Health Science Center, Houston, TX, United States; 3 Division of Developmental Pediatrics and Rehabilitation Medicine, Department of Pediatrics, University of Wisconsin-Madison, Madison, WI, United States; 4 Division of Vascular Neurology, Department of Neurology, McGovern Medical School, University of Texas, Houston, TX, United States; 5 Department of Physical Medicine and Rehabilitation, McGovern Medical School, NeuroRecovery Research Center at TIRR Memorial Hermann, University of Texas, Houston, TX, United States

**Keywords:** childhood stroke, pediatric stroke, tDCS, rehabilitation, neuromodulation

## Abstract

**Background:**

Transcranial direct current stimulation is a non-invasive neuromodulation technique with emerging therapeutic potential in neurodevelopmental conditions. While childhood-onset stroke survivors frequently experience long-term motor impairment, there are very few studies examining the safety and feasibility of transcranial direct current stimulation in this population.

**Objective:**

To evaluate the safety, feasibility, and tolerability of bihemispheric transcranial direct current stimulation paired with occupational therapy in children and adolescents with chronic hemiparesis following childhood-onset arterial ischemic stroke or intracranial hemorrhage.

**Methods:**

In this single-arm, open-label pilot study, five participants aged 6–19 years of age received five daily sessions of transcranial direct current stimulation (sham on day 1, then 0.5–1.5 mA) during structured occupational therapy. Safety and tolerability were assessed through side effect questionnaires, pre-and post-stimulation vital signs, and study completion rates. Secondary exploratory outcomes included arm function measures (Fugl-Meyer Assessment of Upper Extremity, perceived performance and satisfaction) (Canadian Occupational Performance Measure), and gross manual dexterity (Box and Blocks Test).

**Results:**

All participants completed the study with no major adverse events. Mild, self-limited itching or tingling occurred in 40% of sessions and did not limit participation. Four of five participants demonstrated clinically significant improvement on the Fugl-Meyer Assessment of the Upper Extremity at 3-month follow up. Improvements were also observed in the Canadian Occupational Performance Measure and satisfaction scores. One participant with a prior craniectomy tolerated stimulation without adverse events.

**Conclusion:**

tDCS was well-tolerated in children and adolescents with chronic hemiparesis from childhood-onset stroke. These findings support the feasibility of transcranial direct current stimulation in this population and provide early-stage evidence to guide future randomized controlled trials exploring therapeutic applications of neuromodulation in childhood-onset stroke recovery.

**Clinical trial registration:**

## Introduction

Childhood-onset stroke is a leading cause of morbidity in children, with an incidence comparable to childhood brain tumors (Ferriero et al., 2019; Krishnamurthi et al., 2015; National Center for Injury Prevention and Control, CDC, 2018). Unlike perinatal stroke, which occurs in the antenatal or neonatal period, childhood-onset stroke is defined as stroke occurring after the first month of life and accounts for about 50% of pediatric stroke (Ferriero et al., 2019). Although most children survive to adulthood, up to ~75% experience persistent neurologic impairments (Ferriero et al., 2019; Krishnamurthi et al., 2015; Dunbar et al., 2020; Bhatia et al., 2022; Golomb et al., 2007; Billinghurst et al., 2017; Westmacott et al., 2010; Everts et al., 2008; Porcari et al., 2018). Hemiparesis is a common motor sequela, often leading to significant disability, reduced quality of life, and substantial healthcare costs—estimated to exceed $10,000 annually per child (Porcari et al., 2018; Mallick et al., 2016; Goeggel Simonetti et al., 2015; Pulgar et al., 2019). Because stroke occurs during a critical period of brain development, the potential for plasticity-driven recovery is potentially higher than adults (Felling et al., 2020). However, there remains a pressing need for evidence-based interventions that harness this potential to improve functional outcomes.

Transcranial direct current stimulation (tDCS) is a portable, inexpensive, non-invasive neuromodulation technique that applies low-intensity electrical current to modulate cortical excitability (Edwards et al., 2009; Nandi et al., 2022; Hordacre et al., 2021; Ciechanski and Kirton, 2017; Nitsche and Paulus, 2001; Nitsche and Paulus, 2000). The current delivered through tDCS is physiologically small and shown to be safe at intensities up to 4 mA (Nitsche and Bikson, 2017). It does not by itself cause neuronal depolarization. Rather, tDCS is theorized to modulate neuronal activity through alterations in resting membrane potential, altered neurotransmitter signaling, and induced expression of neurotrophic factors which can enhance neuronal plasticity (Philip et al., 2017; Dmochowski et al., 2013). Modeling and safety studies estimate that cortical current density is at least 2 orders of magnitude below neurotoxic thresholds (Sundaram et al., 2009). Across thousands of adults and hundreds of pediatric tDCS study participants, including children with epilepsy and cerebral palsy, serious adverse events have not been reported (Zewdie et al., 2020; Bikson et al., 2016; Hilderley et al., 2025).

Several small trials and multiple systematic reviews and meta-analyses of anodal and bihemispheric tDCS in adults with arm impairment after stroke have shown promising effects of tDCS on upper limb motor recovery (Tedesco Triccas et al., 2016; Butler et al., 2013; Orrù et al., 2020; Li et al., 2018; Shah-Basak et al., 2016), supporting its potential as an adjunct to rehabilitation. Evidence in children however, remains limited. A recent large trial of 83 children with perinatal stroke and unilateral cerebral palsy demonstrated that contralesional cathodal tDCS added to intensive upper extremity therapy did not improve motor outcomes compared to therapy alone (Hilderley et al., 2025). Similarly, a large multicenter trial in adult stroke found no additional benefit of 2 mA or 4 mA bihemispheric tDCS when combined with constraint-induced movement therapy (Schlaug et al., 2025). Both studies affirm the safety and feasibility of tDCS in these populations. However, findings from Nemanich et al. (2023) suggest that variability in the strength of remaining ipsilesional corticospinal tract—identified by the presence or absence of motor-evoked potentials ipsilesional to the paretic hand—may help explain differences in neurophysiologic response to tDCS. Specifically, the authors found increased amplitude of motor evoked potentials after tDCS for hemiparetic participants when the anode was placed on the hemisphere providing corticospinal input to the paretic hand. The authors concluded that differences in corticospinal tract variability between participants after stroke could be a key determinant of individual motor recovery potential with tDCS.

These findings underscore the complexity of applying neuromodulation across diverse populations. Children with childhood-onset stroke, as distinct from perinatal stroke or adult-onset stroke, represent a unique group in whom brain development, injury timing, and neuroplastic potential may influence responsiveness to stimulation in different ways. Additionally, childhood-onset stroke is divided by heterogenous lesion types, post-surgical changes to skull morphology and thickness, and varying degrees of corticospinal tract preservation, all of which may affect feasibility, efficacy, and outcome trajectories differently from other populations. To date, the only prior publication of tDCS in childhood-onset stroke is from a single case report (Ciechanski et al., 2020). A recent clinical trial of tDCS did include participants with stroke or traumatic brain injury before 1 year of age (Nemanich et al., 2023). However, this prior study focused primarily on perinatal and early brain injury, and did not include participants with intracranial hemorrhage, post-craniectomy anatomy, or late-childhood onset stroke.

In this context, the TOlerability of transcranial direct current stimulation in Pediatric Stroke Survivors (TOPSS) study was conducted. This was an open-label, single-arm pilot trial designed to evaluate the feasibility, safety, and tolerability of bihemispheric tDCS paired with occupational therapy in children and adolescents with chronic hemiparesis from childhood-onset stroke. We hypothesized that tDCS would be well tolerated, including in participants with prior craniectomy, and no serious adverse events would occur. This small feasibility study was designed to provide a foundation for future randomized controlled clinical trials in this critically understudied patient population.

## Methods

### Ethics and approval

Tolerability of transcranial direct current stimulation in Pediatric Stroke Survivors (TOPSS) was a single-center, unblinded tolerability and feasibility pilot study. The primary question was whether the intervention was safe, tolerable and feasible among childhood-onset stroke survivors participating in the study. The study protocol was approved by the UTHealth Institutional Review Board, protocol number HSC-MS-23-0092. The study was conducted in accordance with their recommendations. The study was registered at clinicaltrials.gov (NCT05812794). Written informed consent was obtained from the guardians of participating children and participants aged 18 or older. Verbal and written assent was obtained when applicable for all participants under the age of 18. The study was overseen by a Data Safety and Monitoring Board (DSMB) consisting of a pediatric physiatrist and pediatric neurologist.

### Study design and recruitment

**Figure 1:**
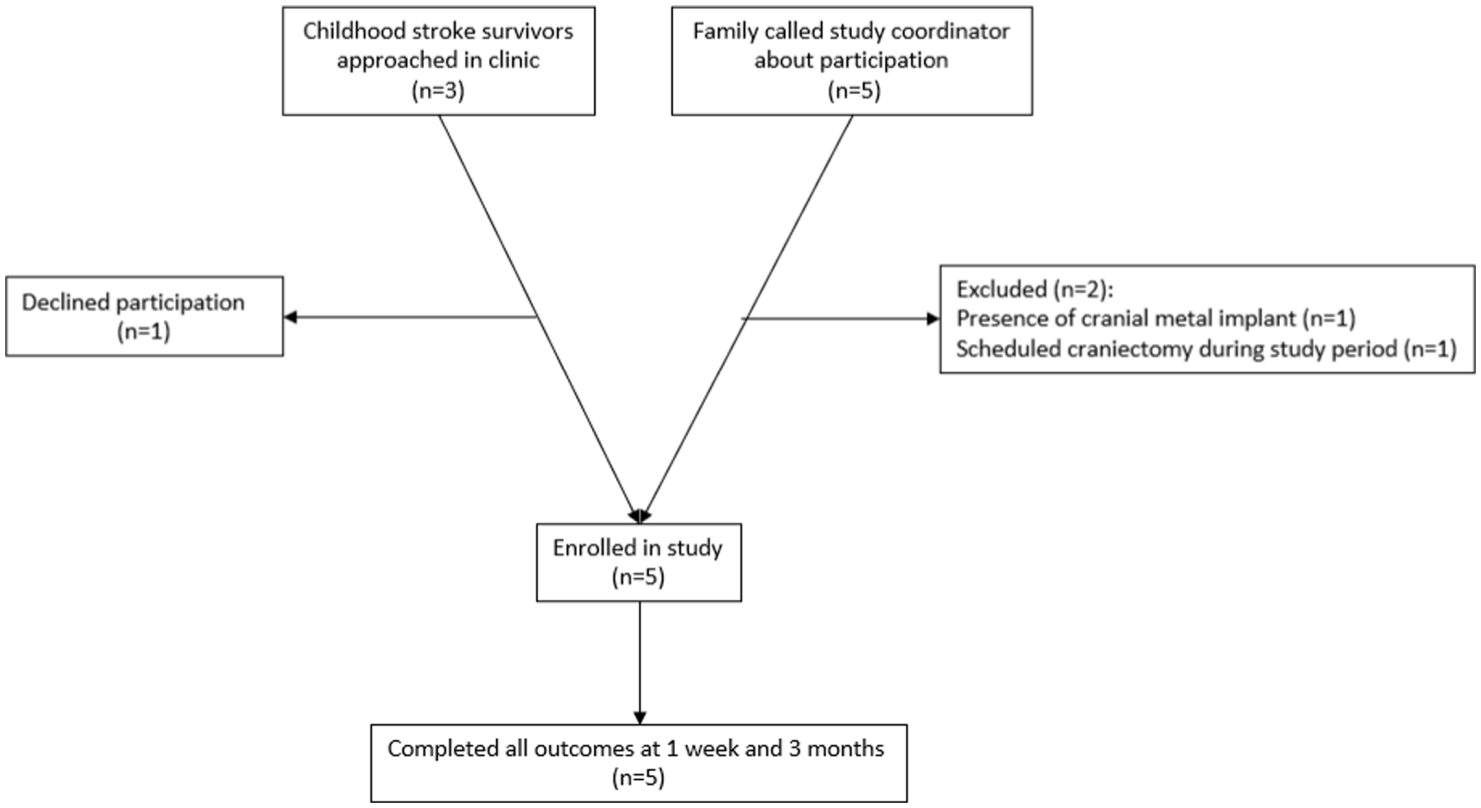
Schematic of screened and enrolled participants.

Participants were recruited through the UTHealth Pediatric Stroke Clinic and through participation flyers distributed to local advocacy and support organizations. Inclusion criteria were: (1) Clinical diagnosis of childhood-onset arterial ischemic or hemorrhagic stroke (2) Age 5–19 years (3) At least 3 months from stroke diagnosis (4) chronic hemiparesis due to the childhood-onset stroke, with a Fugl-Meyer Assessment of the Upper Extremity Assessment score ≤58. Exclusion criteria included (1) seizure within the past 6 months (2) cranial metal implants contraindicated to tDCS, such as programmable ventriculoperitoneal shunt, (3) inability to participate in 2-h rehabilitation therapy sessions due to medical fragility (4) craniectomy without bone flap reimplantation, or if the bone flap has been reimplanted <6 months prior to participation. Each study participant and/or their legal guardian, as applicable, completed a medical screening form prior to participation. As this was an open-label pilot study, no power calculation was done prior to participation. A feasibility sample of 5 participants was recruited. A recruitment flowchart is shown in Figure 1. One potential participant declined participation after discussing consent for the study due to concern regarding the potential of side effects associated with the experimental device.

### Outcome measures

Participants were assessed at 3 separate time points by the treating therapist and the study PI. The baseline assessment was completed 1 week before the intervention. A follow up assessment was performed the week after the intervention, and a final assessment was performed 3 months after the intervention.

The primary objective measures were safety and tolerability which were assessed with percent study completion, participant tolerability questionnaires, and pre-and post-stimulation vital signs. Questionnaires were adapted from previously published safety studies in cerebral palsy (Ciechanski and Kirton, 2017; Gillick et al., 2015; Gillick et al., 2018). Participants were asked a series of questions regarding side effects prior to stimulation, during stimulation, and immediately after stimulation. These questions were verbally administered by a trained investigator using developmentally appropriate language as needed to for comprehension across all participants in the study. Questions asked included the presence and intensity of headache, tingling, itchiness, nausea, dizziness, cognitive changes, and other potential side effects of non-invasive neuromodulation. The adverse event monitoring approach, risk mitigation protocol, and side-effect questionnaire were adapted from a prior published set of safety questionnaires outlined by Gillick et al. (2018). The entire questionnaire and safety sheet is available in Supplementary material 1. Blood pressure and pulse rate were measured by the research nurse immediately pre-stimulation, post-stimulation, and at the end of each stimulation session using an automated vital sign machine.

Measures of arm function, satisfaction, and overall level of neurologic impairment during the pre-, post-, and 3-month follow up assessments were obtained. Arm function was measured using the Fugl-Meyer Assessment of the Upper Extremity by the treating therapist during the assessment visits. The Fugl-Meyer Assessment of the Upper-Extremity (FMA-UE) is a functional assessment of the arm that is scored from 0 (complete hemiplegia) to 66 (normal arm function) and can typically be obtained in 30 min. It is one of the most commonly used outcome measures in adult stroke literature, and children as young as 5 have been demonstrated to reliably perform the test (Fasoli et al., 2009). The Canadian Occupational Performance Measure (COPM), a subjective measure of task-specific performance and satisfaction graded by participants and their parents, was also obtained. The Box and Blocks test, a widely used, simple 2-min test of moving blocks from one side of a barrier to another using one of the upper extremities, was also obtained. The FMA-UE, COPM, and Box and Blocks test were done by the treating therapist. One of the study physicians obtained a pediatric stroke outcome measure (PSOM) at each of the assessment visits. The Pediatric Stroke Outcome Measure is a physician-administered rating scale of global function scored from 0 (no deficit) to 10 (profound deficit, immobile and non-responsive). The pediatric stroke outcome measure has been validated for classifying global neurological deficit in pediatric stroke (Slim et al., 2020).

### Statistical analysis

Primary and secondary outcomes were summarized using descriptive statistics, including medians, ranges, and proportions where applicable.

### Intervention: tDCS paired with rehabilitation therapy

tDCS sessions occurred in the University of Texas Professional Building in the Center for Treatment of Pediatric Neurodegenerative Diseases clinical research suite between 10 AM-4 PM based on participant and therapist availability. All study participants received 5 sessions of tDCS lasting for 20 min each (100 min total). Five sessions occurred once daily for a five-day period. The stimulator used was the Soterix 1×1 tDCS device.

**Figure 2:**
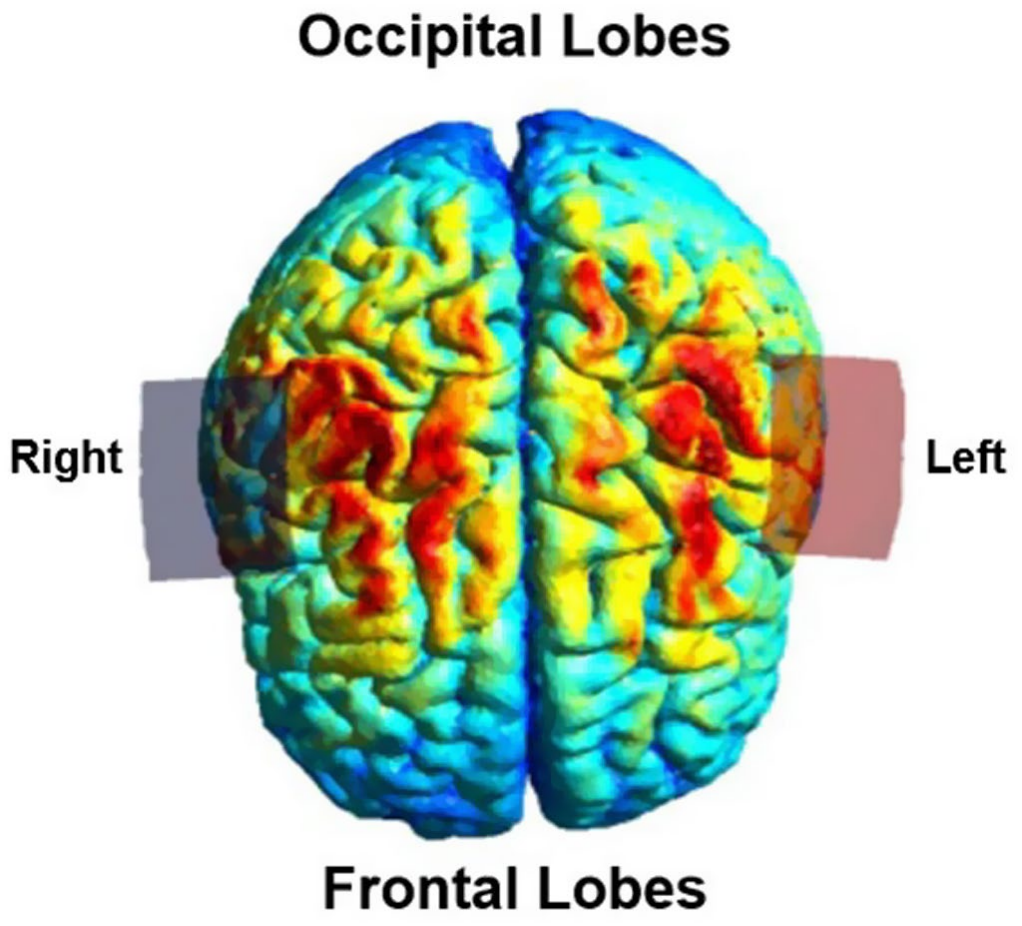
Simulation of non-invasive brain stimulation (SimNIBS 4.5) (Thielscher et al., 2015) depiction of the electric field distribution using a bihemispheric transcranial direct current stimulation montage with electrodes placed at C3 and C4 according to the international 10/20 EEG system. The electrode placement was adapted to each participant’s lesion side (right or left hemisphere). The figure represents the modeled electric field for a representative example montage, visualized from a top down perspective. The red electrode (anode) is over the C3 position.

Before participating in therapy, each participant completed a pre-therapy ‘safety checklist’. For each tDCS session, 5 cm × 5 cm sponge electrodes were moistened using 5 mL of normal saline. The sponges were placed in a montage often referred to as the “bihemispheric” montage as shown in Figure 2 (Waters-Metenier et al., 2014; Shinde et al., 2021). This montage, with the anode over the lesioned motor cortex (C3 or C4) and cathode over the contralesional motor cortex, was chosen based on results from prior adult upper extremity stroke recovery trials (Tedesco Triccas et al., 2016). 5 cm × 5 cm sponge electrodes were chosen to accommodate a wide range of available head sizes, including smaller head circumferences expected for younger participants. Electrodes were placed as follows; the study principal investigator and research nurse measured head circumference, auricular to auricular, and nasion to inion distances. Using these measurements and the standard international 10/20 EEG system, the Cz, C3, and C4 positions on the head were marked using a washable marker. After this the principal investigator and research nurse placed the anode over the lesioned hemisphere, either at the C3 position if the stroke was left sided or C4 if the stroke was right sided. The cathode was placed at the contralateral C3 or C4 position. Sponges were secured in place using the Soterix easy-strap system using an appropriately sized strap based on the participants head circumference. After contact quality was verified on the device by the principal investigator, the current was ramped up over 30 s for each study session per standard Soterix 1×1 device function. Stimulation time was set to 20 min per session. For sham stimulation, the current ramped down over 30 s after ramping up to 0.5 mA. The amplitude of stimulation was increased daily as follows: sham stimulation on day 1, 0.5 mA on day 2, 1.0 mA on day 3. Current remained at 1.0 mA for days 4 and 5 for participants aged 5 to 12 years. Participants aged 13–19 years had stimulation increased to 1.5 mA on days 4 and 5. This staged protocol was designed to assess tolerability and participant comfort at different levels, allowing intra-participant comparison of endorsed side effects at different stimulation levels and to ensure safety in this population with variable cranial anatomy and surgical history. Our choice of maximum intensity of 1 mA in children and 1.5 mA in adolescents was informed by electric field modeling studies finding that younger children likely experience higher cortical electric field strengths at a given stimulation intensity than adults (Carlson et al., 2023).

Participants engaged in skilled therapy intervention with a licensed occupational therapist experienced in stroke rehabilitation. Therapy was conducted for 2 h total during the 5 consecutive treatment days. Participants received tDCS during the first 20 min of therapy. Participants completed the remaining 100 min of therapy after stimulation concluded. Interventions targeting motor recovery of the impaired upper extremity were individualized based on activities identified as meaningful to each participant during the baseline assessment. Goal-directed therapy sessions focused on motor learning, bilateral training, forced use, strengthening, range of motion, and coordination interventions, ultimately leading to functional task practice. Therapy was personalized using task-specific goals identified during the baseline assessment. Examples of targeted movements include functional reach in multiple planes in preparation for washing/brushing hair, donning/doffing a shirt, and playing sports; whole-hand grasp/release in preparation for doing dishes, laundry, and cooking; and controlled thumb movements in preparation for playing video games.

**Figure 3:**
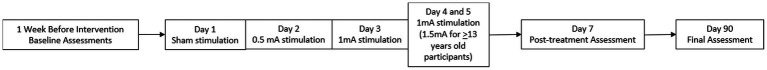
Study timeline.

A visual representation of the timeline of study procedures is demonstrated in Figure 3.

### Data analysis

All data was entered into a Research Electronic Data Capture (REDCap) database (Harris et al., 2009). Descriptive statistics were used to report primary and secondary outcomes.

## Results

During the enrollment period, participation was discussed with 8 families. 1 family declined participation due to concerns about potential side effects. 2 were excluded due to the presence of contraindications (Figure 1).

### Primary outcomes

Baseline participant demographics are presented in Table 1. The median age of participation was 15 years. Participants represented a heterogeneous cohort, including three with arterial ischemic stroke and two with intracranial hemorrhage. Stroke etiologies included traumatic dissection, cerebral arteriovenous malformation rupture, meningitis related coagulopathy, and aneurysm rupture. Sex, race and ethnicity were diverse in this cohort. The median time from stroke onset to beginning the study was 1 year. The median PSOM score was 1, and the PSOM did not change for any of the participants during the study period. All participants were able to complete the study. There were no episodes of tachycardia, bradycardia, hypertension or hypotension pre or post therapy. Secondary outcome measures including the Fugl-Meyer Assessment of the Upper Extremity (FMA-UE), Canadian Occupational Performance Measure (COPM), and Box and Blocks assessment are shown in Table 2.

**Table 1:** Baseline demographics.

Characteristic	Participant 1^a^	Participant 2	Participant 3	Participant 4	Participant 5^b^
Age during participation (years)	13	17	15	6	19
Age at stroke (years)	12	17	0.5	5	16
Sex	Female	Male	Female	Male	Male
Race^c^	Asian	Black	White	White	White
Ethnicity	Non-Hispanic	Hispanic	Non-Hispanic	Non-Hispanic	Hispanic
Type of stroke	Intracranial Hemorrhage	Arterial Ischemic	Intracranial Hemorrhage	Arterial Ischemic	Arterial Ischemic
Etiology	Aneurysm Rupture	Traumatic Dissection	Meningitis and Disseminated Intravascular Coagulation	Focal Cerebral Arteriopathy	Complication of Cerebral Arteriovenous Malformation Embolization
Dominant hemisphere	Left	Right	Right	Left	Left
Arm affected	Left	Left	Right	Left	Right
History of seizures at stroke presentation	Yes	No	Yes	Yes	Yes
Diagnosis of epilepsy	No	No	Yes	No	Yes
Antiepileptics	Levetiracetam	0	Levetiracetam	0	Levetiracetam, Clobazam, Oxcarbazepine
PSOM Total	2	1	4	1	2

a Participant 1 had right craniectomy at stroke presentation. Had cranioplasty with bone flap reattachment 10 months prior to study participation.

b Participant 5 had a history of multiple prior endovascular arterial embolizations, but no neurosurgical history, such as craniotomy or craniectomy. No other participants had histories of neurosurgical interventions or endovascular intervention (e.g., ventriculoperitoneal shunt placement or thrombectomy).

c Race and ethnicity are defined using US Census Bureau Definitions.

**Table 2:** Secondary outcome measures.

Measure/Timepoint	Participant 1	Participant 2	Participant 3	Participant 4	Participant 5
FMA-UE					
Baseline	32	48	35	37	32
Post-therapy	38	52	35	43	41
Three month follow up	37	52	35	44	42
COPM-performance					
Baseline	4	2.8	2.2	6.4	4.2
Post-therapy	9.4	5.8	2.2	7	3.4
Three month follow up	9.2	6.4	2.2	7.2	3.4
COPM-satisfaction					
Baseline	4.4	1.8	4	7	3.2
Post-therapy	10	7.4	4	6.8	3.2
Three month	10	5.2	4	6.6	3.2
Box and blocks—affected side^a^					
Baseline	1	21	5	7	14
Post-therapy	2	30	11	7	12
Three month	0	30	12	7	13
Box and blocks—unaffected side					
Baseline	54	62	31	29	61
Post-therapy	64	67	26	34	60
Three month follow up	63	71	32	37	54

a Box and blocks scores are reported in blocks/min.

Blood pressure and pulse measurements were within normal age-adjusted limits for all participants for all pre-and post-therapy measurements with no episodes of tachycardia, bradycardia, hypertension or hypotension (Sepanski et al., 2018). A sample safety questionnaire is provided in Supplementary material 1. Complete data for all survey responses and vital sign measurement in every session by all participants are present in Supplementary material 2. A complete print out of every vital sign measurement and side effect endorsed are available upon request. Participants endorsed itchiness or tingling during 40% of tDCS sessions, which were benign and self-limiting. Overall, participants endorsed any side effects as follows: 80% of participants on day 1 (sham stimulation), 40% on day 2, 40% on day 3, 20% on day 4, and 40% on day 5. Four participants endorsed itchiness or tingling on 2 or fewer days, and 1 participant endorsed tingling and itchiness with every stimulation day. The participant who did endorse daily itchiness and tingling did have a history of craniectomy with bone flap replacement 10 months prior to participation in the study and associated skin sensitivity on the affected side of the head. Notably, this participant endorsed a similar degree of itching and tingling on every therapy day of participation, including the sham-stimulation day. Itchiness and tingling did not limit participation for any participants.

### Secondary outcomes

**Figure 4:**
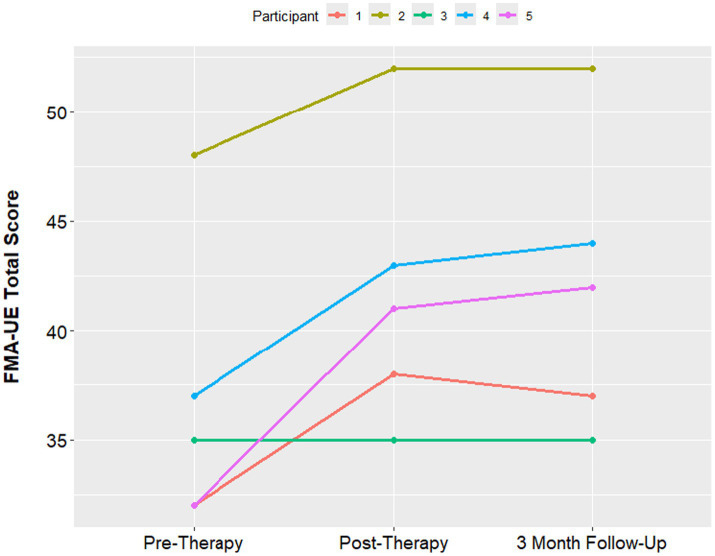
Median FMA-UE at baseline, post-therapy visit and 3-month follow up visit.

The median Fugl-Meyer UE score was 35 at the pre-therapy assessment, with a minimum of 32 and maximum of 48. At the 1-week post therapy assessment visit, the median FMA-UE score improved to 41, with a minimum of 35 and maximum of 42. The median FMA-UE improved to 42 at the 3 month follow up, with a minimum of 37 and maximum of 44. An improvement of 5 points in the FMA-UE is often considered to be the minimum clinically significant difference (Hiragami et al., 2019). 4 out of 5 participants had a clinically significant increase in the Fugl Meyer score. The one participant who had no improvement in the Fugl-Meyer also had the longest time since stroke at 15 years (Figure 4). PSOM scores remained stable from baseline to 3-month follow up in all participants.

**Figure 5:**
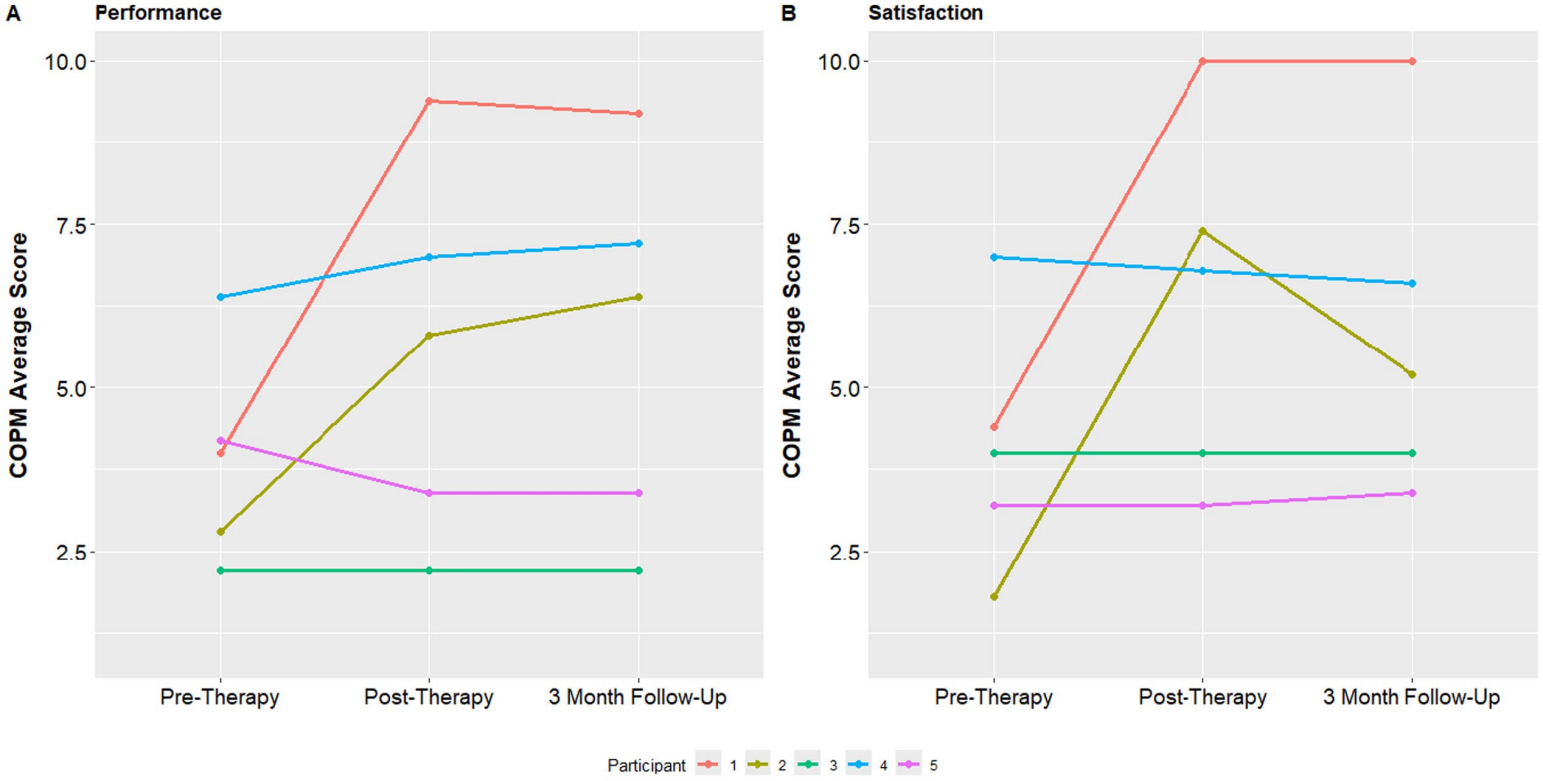
COPM performance and satisfaction at baseline, post-therapy visit and 3-month follow up visit.

The median COPM performance score was 4 at baseline with a minimum of 2.8 and maximum of 4.2. This score improved to 5.8 at the post assessment visit (minimum 3.4, maximum 7.0) and to 6.4 at 3 month follow up (minimum 3.4, maximum 7.2). The COPM satisfaction score was 4.0 at baseline with a minimum of 3.2 and maximum of 4.4. This median satisfaction score was 6.8 at the 1 week follow up (minimum 4.0, maximum 7.4) and 5.2 at the 3 month follow up assessment (minimum 4.0, maximum 6.6) (Figure 5).

**Figure 6:**
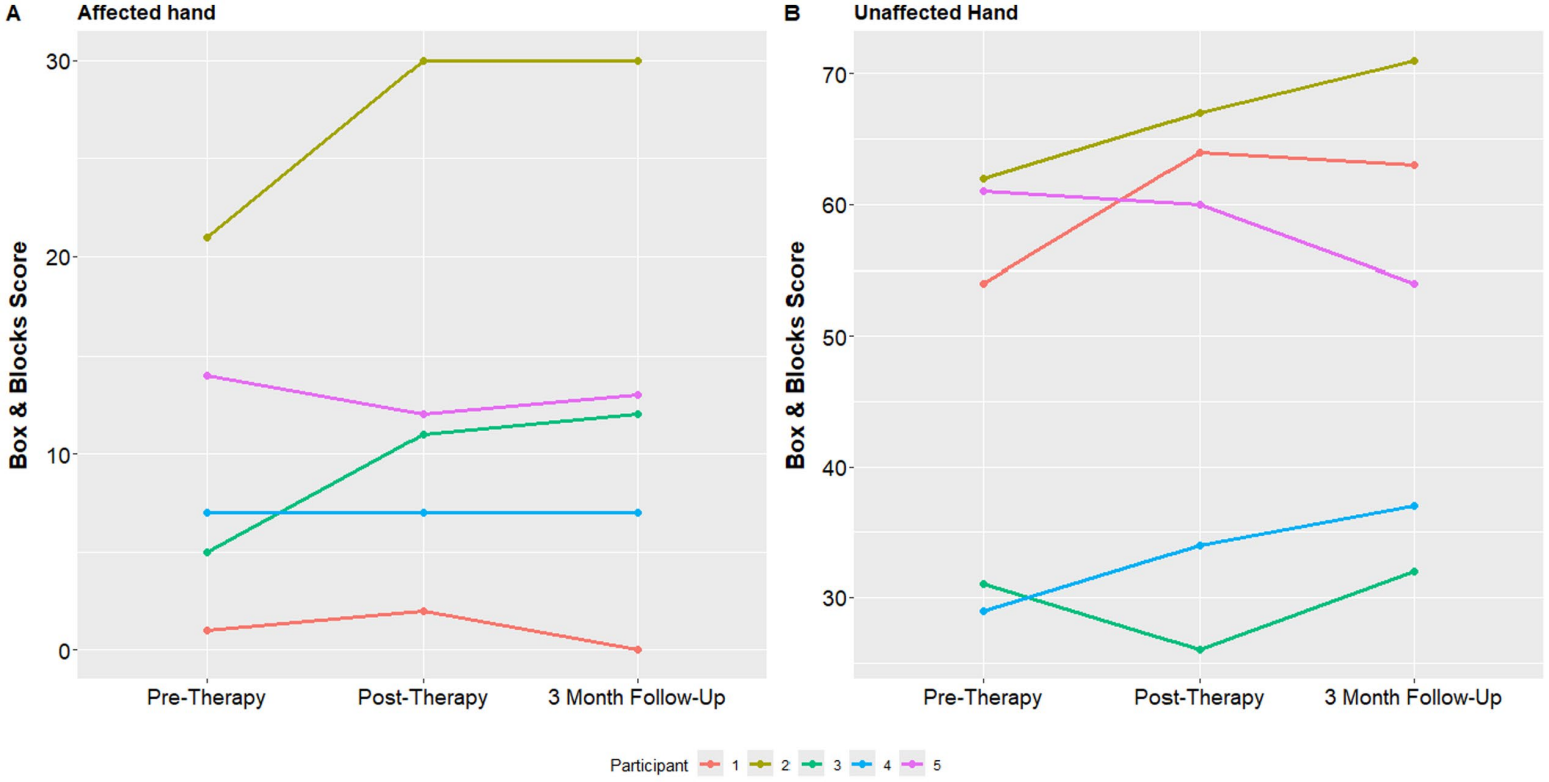
Median box and blocks score over time.

Finally, individual mean Box and Blocks scores at all 3 study assessments with both the affected and unaffected hand are shown in Figure 6. The median box and blocks score of the affected hand at the initial visit was 7, which improved to 11 at the one-week follow up visit and 12 at the 3 month follow up visit.

## Discussion

This is the first multi-participant pilot study of tDCS in children with chronic arm impairment from childhood-onset stroke (Ciechanski et al., 2020). Bihemispheric tDCS applied for five sessions was well-tolerated by all participants. There were no major adverse events, and the most common reported side effects were mild itching or tingling, which did not limit participation and was self-limiting. All enrolled participants were able to complete the study. Importantly, given the exploratory nature of this feasibility trial, children with both ischemic stroke and intracranial hemorrhage were included in the trial to reflect the clinical heterogeneity encountered in real-world pediatric stroke rehabilitation. One participant had a history of craniectomy and had bone flap replacement 10 months prior to participating in the study. This is important as many previous tDCS studies in children have not mentioned inclusion of children with prior craniectomy, which can theoretically alter the amount of current entering the brain. However, prior simulation work has indicated the amount of current entering the brain in patients with healed skull defects remains at least 2 orders of magnitude below theoretically damaging levels to neurons (Datta et al., 2010). Due to the small sample size and feasibility design of this study, no inferential statistical analyses were performed. This study is meaningful as an early step in extending non-invasive neuromodulation research for children living with neurologic impairment from childhood-onset stroke, which has not been done previously.

For secondary measures, we assessed upper extremity function and performance. Fugl-Meyer of the Upper Extremity scores improved an average of 7 points at the 3 months post therapy assessment, and COPM performance scores improved an average of 2.4. Both of these improvements are considered clinically significant (Hiragami et al., 2019; McColl et al., 2023). As this pilot study was open label with no sham group, caution must be used in extrapolating any conclusions from the improvements in upper extremity function and performance found in this study. We cannot say whether the limited amount of tDCS these participants received contributed to these improvements. However, it does indicate a significant potential for recovery in response to rehabilitation therapy in this population of participants with childhood-onset stroke in the chronic phase of recovery. In this study, clinically significant gains in arm function were present in 4 out of 5 participants after 10 h of 1-on-1 rehabilitation therapy. Notably, the participant with the smallest change in the FMA-UE score also had the greatest time between stroke onset and starting therapy (15 years). As the intervention was well tolerated and feasible to recruit for, a double blinded, randomized controlled trial of tDCS in this patient population with higher stimulation intensities up to 2 mA is currently underway. This ongoing trial of tDCS in participants with childhood-onset stroke and hemiparesis (The TOPSS 2 Trial—NCT06358690) will generate estimates of efficacy of tDCS in subjects with childhood-onset stroke.

In addition to the large recovery potential of childhood-onset stroke participants, it is important to note the unmet needs of recovery research in childhood-onset stroke. Recruitment opened in May 2023 and was closed in July 2023, which was much faster than we had previously anticipated. Childhood-onset stroke has left thousands of children and adolescents in the world with chronic neurologic impairment, and many of them will have moderate to severe upper extremity motor impairment. Hemiparesis impacts quality of life and workforce participation, and improving the functional abilities of the thousands of children with chronic arm impairment could be profoundly beneficial to both them, their families, and society as a whole. Future studies in tDCS for recovery in childhood-onset stroke should include similar safety standards and study criteria that were used in prior studies of tDCS in cerebral palsy, including perinatal stroke. Additionally, future research in this field should include the natural history of recovery in childhood-onset stroke, validation of additional outcome measures, such as the Fugl-Meyer Assessment of the Upper Extremity, and biomarkers investigating the mechanism of action of tDCS in childhood-onset stroke.

There are several important limitations to this study. This small feasibility trial was not designed to prove efficacy. Instead, we investigated the feasibility and tolerability of doing open-label tDCS in childhood-onset stroke survivors. As this study had unblinded participants and treating staff, no conclusions about the effect of tDCS to improve motor recovery in childhood-onset stroke should be made from this trial. Additionally, the enrolled sample was skewed towards adolescents with chronic stroke, which may limit generalizability to younger childhood-onset stroke survivors. While this study used conservative stimulation intensities (maximum of 1.5 mA in adolescents), further research is needed to determine the tolerability and potential efficacy of higher stimulation doses (2 mA or potentially higher) in younger children with childhood-onset stroke.

## Conclusion

tDCS in the bihemispheric montage at up to 1.5 mA was well tolerated in this small pilot study of tDCS feasibility and tolerability in study participants with hemiparesis from childhood-onset stroke. While most participants endorsed some mild and self-limiting side effects such as itching or tingling, there were no side effects that limited participation. There were no major adverse events, and four out of five participants had clinically significant improvements in the Fugl-Meyer Assessment of the Upper Extremity. Future research should include randomized, blinded studies to determine whether tDCS can augment motor recovery in childhood-onset stroke.

## Data Availability

The original contributions presented in the study are included in the article/Supplementary material, further inquiries can be directed to the corresponding author.
