# Correction: TOPSS: TOlerability of transcranial direct current stimulation in Pediatric Stroke Survivors

**DOI:** 10.3389/fnhum.2025.1739609

**Published:** 2025-11-21

**Authors:** Stuart Fraser, Anna Clearman, Melika Abrahams, Bernadette Gillick, Tia Lal, Sean Savitz, Nuray Yozbatiran

**Affiliations:** 1 Division of Child and Adolescent Neurology, Department of Pediatrics, McGovern Medical School, University of Texas, Houston, TX, United States; 2 Institute for Stroke and Cerebrovascular Disease, University of Texas Health Science Center, Houston, TX, United States; 3 Division of Developmental Pediatrics and Rehabilitation Medicine, Department of Pediatrics, University of Wisconsin-Madison, Madison, WI, United States; 4 Division of Vascular Neurology, Department of Neurology, McGovern Medical School, University of Texas, Houston, TX, United States; 5 Department of Physical Medicine and Rehabilitation, McGovern Medical School, NeuroRecovery Research Center at TIRR Memorial Hermann, University of Texas, Houston, TX, United States

**Keywords:** childhood stroke, pediatric stroke, tDCS, rehabilitation, neuromodulation

The funder National Institute of Health, Grant Number 5K12NS098483-08 was erroneously omitted.

The original version of this article has been updated.

