# Supplementary material for: TOPSS: TOlerability of transcranial direct current stimulation in Pediatric Stroke Survivors
Source: Front Hum Neurosci. 2025 Aug 5;19:1629499. doi: 10.3389/fnhum.2025.1629499 (PMC12362507; doi:10.3389/fnhum.2025.1629499)
Supplement: Supplementary file 1 [file Table_1.DOCX]

TOPSS Study

**Risks and Mitigation of Risks Transcranial Direct Current Stimulation (tDCS)**

Subject ID:_______________

| **Study Procedure** | **Anticipated Risks** | | **Risk Mitigation** |
| --- | --- | --- | --- |
| tDCS | Burn- Electrolysis | | Ensure proper electrode contact with skin |
| tDCS | Stimulation over broken skin, reduced resistance | | Assess skin integrity, avoid placement of electrodes over recent shaving, skin defects |
| tDCS | Stimulation over conductive implants | | Screen appropriately for exclusion criteria of implants |
| tDCS | Itching, Tingling, Burning Sensation in the area of the electrodes | | Ensure proper contact of surface electrodes with skin. Maintain current dosage within low-range of researched dosages. Ensure that electrode sponges are properly sanitized and that saline solution is appropriately employed. |
| tDCS | Headache | | Ensure that headband securing electrodes is in proper placement, yet not to the level of impingement of scalp area. Maintain current dosage within low range of delivery. |
| tDCS | Pain-Neck, Scalp | | Ensure that electrodes are in proper contact with  skin and adjust head position as needed for comfort. |
| tDCS | | Skin Redness | Ensure proper electrode position and proper level of moisture to even stimulation across the electrode |

Investigator Completing Screening:

Name (Print) : _______________________________

Signature:___________________________________ Date:_______________________________

**tDCS Stimulation Monitoring Sheet**

Participant ID: PI: Stuart Fraser, MD

Visit Number:

Date:

| **Visit Date:** | | **PRE tDCS** Time: Investigator: | | **Mid-Intervention**  **Time:** | **POST** Time: Investigator: | |
| --- | --- | --- | --- | --- | --- | --- |
| **How are you feeling overall right now?** | Participant |  | | **How is the stimulation feeling right now?** |  | |
|  | Caregiver |  | |  |  | |
| **“Right now, do you feel you have or are……?”** | | **Value**  **(per child)**  1 absent  2 mild  3 moderate 4 severe | **Relation (per investigator)**  1 unrelated  2 unlikely  3 possible 4 probable  5 definite |  | **Value**  **(per child)**  1 absent  2 mild 3 moderate  4 severe | **Relation (per investigator)**  1 unrelated  2 unlikely  3 possible 4 probable  5 definite |
| Headache | |  |  |  |  |  |
| Unusual feelings on the skin of your head | |  |  |  |  |  |
| Neck pain | |  |  |  |  |  |
| Tingling | |  |  |  |  |  |
| Itchiness | |  |  |  |  |  |
| Sleepiness | |  |  |  |  |  |
| Difficulty paying attention | |  |  |  |  |  |
| Unusual feelings, attitude, emotions | |  |  |  |  |  |
| Tooth pain | |  |  |  |  |  |
| Change in hearing | |  |  |  |  |  |
| Nausea/Sick to Stomach | |  |  |  |  |  |
| Unusual twitches or movements in muscles | |  |  |  |  |  |
| Dizziness | |  |  |  |  |  |
| Anxious/Worried/Nervous | |  |  |  |  |  |
| Forgetful | |  |  |  |  |  |
| Difficulty with your balance | |  |  |  |  |  |
| Change in movement in your stronger hand | |  |  |  |  |  |
| Abnormal sleep last night | |  |  |  |  |  |
| Seizure within the last 24 hours | |  |  |  |  |  |
| Other: | |  |  |  |  |  |

Add comments in table if needed

**Pre-tDCS:**

**Time:**

**Investigator:**

**Vital Signs:**

**Pulse:__________________ Blood Pressure:______________**

**OT Assessment**

**Peg-Board Score Right Hand:­­­­­_________**

**Peg-Board Score Left Hand:___________**

**Post-tDCS:**

**Time:**

**Investigator:**

**Vital Signs:**

**Pulse:__________________ Blood Pressure:______________**

**OT Assessment**

**Peg-Board Score Right Hand:­­­­­_________**

**Peg-Board Score Left Hand:___________**

**End of Therapy Session:**

**Time:**

**Investigator:**

**Vital Signs:**

**Pulse:__________________ Blood Pressure:______________**

**OT Assessment**

**Peg-Board Score Right Hand:­­­­­_________**

**Peg-Board Score Left Hand:___________**

**Investigator Name (Print):_________________________________**

**Signature:_______________________________ Date:______________________________**
